# Elevated expression of pancreatic adenocarcinoma upregulated factor (PAUF) is associated with poor prognosis and chemoresistance in epithelial ovarian cancer

**DOI:** 10.1038/s41598-018-30582-8

**Published:** 2018-08-15

**Authors:** Chel Hun Choi, Tae Heung Kang, Joon Seon Song, Young Seob Kim, Eun Joo Chung, Kris Ylaya, Seokho Kim, Sang Seok Koh, Joon-Yong Chung, Jae-Hoon Kim, Stephen M. Hewitt

**Affiliations:** 1 Experimental Pathology Laboratory, Laboratory of Pathology, Center for Cancer Research, National Cancer Institute, National Institutes of Health, Bethesda, MD 20892 USA; 2 Department of Immunology, College of Medicine, Konkuk University, Chungju, 27478 Republic of Korea; 3 Radiation Oncology Branch, Center for Cancer Research, National Cancer Institute, National Institute of Health, Bethesda, MD 20892 USA; 4 Department of Obstetrics and Gynecology, Gangnam Severance Hospital, Yonsei University College of Medicine, Seoul, 06273 Republic of Korea; 5 Departments of Obstetrics and Gynecology, Samsung Medical Center, Sungkyunkwan University School of Medicine, Seoul, 06351 Republic of Korea; 6 Department of Pathology, Asan Medical Center, University of Ulsan College of Medicine, Seoul, 05505 Republic of Korea; 7 Aging Research Institute, Korea Research Institute of Bioscience and Biotechnology, Daejeon, 34141 Republic of Korea; 8 Department of Biological Sciences, Dong-A University, Busan, 49315 Republic of Korea

## Abstract

Pancreatic adenocarcinoma upregulated factor (PAUF) is a ligand of toll-like receptors (TLRs) and has been reported to be involved in pancreatic tumor development. However, the significance of PAUF expression in epithelial ovarian cancer remains unclear. We aimed to investigate the possible clinical significance of PAUF in epithelial ovarian cancer. We examined the link between PAUF and TLR4 in ovarian cancer cell lines. Recombinant PAUF induced cell activation and proliferation in ovarian cancer cell lines, whereas PAUF knockdown inhibited these properties. Subsequently, we assessed PAUF and TLR4 expression by immunohistochemistry on tissue microarray of 408 ovarian samples ranging from normal to metastatic. PAUF expression positively correlated with TLR4 expression. Overexpression of PAUF was associated with high-grade tumor (*p* = 0.014) and chemoresistant tumor (*p* = 0.017). Similarly, high expression of TLR4 correlated with advanced tumor stage (*p* = 0.002) and chemoresistant tumor (*p* = 0.001). Multivariate analysis indicated that PAUF^high^, TLR4^high^, and PAUF^high^/TLR4^high^ expression are independent prognostic factor for progression-free survival, while TLR4^high^ and PAUF^high^/TLR4^high^ expression were independent prognostic factors for overall survival. Our results suggest that PAUF has a role in ovarian cancer progression and is a potential prognostic marker and novel chemotherapeutic target for ovarian cancer.

## Introduction

Ovarian cancer is the leading cause of gynecologic cancer-related deaths in the United States, accounting for 13,850 deaths annually^1^. Despite significant advances in diagnosis and treatment, more than 70% of affected women are diagnosed with advanced disease, and ovarian carcinoma remains the most lethal gynecologic tumor^2–4^. This high mortality is attributed in part to the lack of reliable early detection method and inadequate primary treatment regimens resulting in rapid tumor recurrence, which is typically less responsive to current chemotherapy strategies, resulting in poor patient outcome^5,6^. Thus, there is a great need for research studies into the molecular pathogenesis of ovarian cancer to facilitate screening and to encourage the development of novel therapeutic strategies to prevent disease recurrence.

It is well appreciated that inflammation resulting from chronic infection and irritation is an important factor in cancer tumorigenesis and progression^7,8^; in particular, numerous inflammatory cells and various cytokines are present in ascites fluid and ovarian cancer tissue^9,10^. Furthermore, prior studies demonstrated that inflammatory mediators and cytokines produced by activated immune cells promote ovarian tumorigenesis and cancer progression^11,12^. Although there is increasing evidence of the importance of inflammation in ovarian cancer progression, the source and target of the inflammatory signals are unknown. Among molecules involved in cancer-related inflammatory responses, toll-like receptors (TLRs) are pattern recognition receptors that have a crucial role in innate inflammation and innate immunity^13–17^. As potential activators of NF-kB, TLRs are considered to be the gateway to inflammation and tumorigenesis^18,19^. Furthermore, TLRs have also been detected in several malignant epithelial tumors^20^ and have been shown to promote cell proliferation, inhibit apoptosis, and lead to cell migration, invasion, and angiogenesis^19^.

Pancreatic adenocarcinoma up-regulated factor (PAUF) is a novel tumor-specific protein^21^ that plays an important role in carcinogenesis and metastasis in several types of cancer^21–23^. It has been associated with poor outcomes in cervical cancer^23^. PAUF promotes both angiogenesis and vascular permeability in a mouse pancreatic cancer model^24^. Furthermore, PAUF has been reported to be an endogenous ligand for TLR4 and can lead to activation of extracellular signal-regulated kinase (ERK), c-Jun N-terminal kinase (JNK), protein kinase B (AKT)^22^, and p-38 of the innate immunity TLR signaling pathway without activating NF-*k*B in a human leukemia cell line (THP-1)^25^. Thus, we hypothesized that the PAUF/TLR4 signaling pathway may play a role in the development of ovarian cancer and potentially a novel target for treatment. The aim of this study was therefore to examine the potential association between PAUF and TLR4 in ovarian cancer progression using ovarian cancer cell lines. Additionally, we assessed the clinical applicability of PAUF and TLR4 expression as a prognostic and predictive biomarker in ovarian cancers.

## Results

### PAUF is linked to TLR4-mediated signaling and cell proliferation in ovarian cancer cell lines

**Figure 1:**
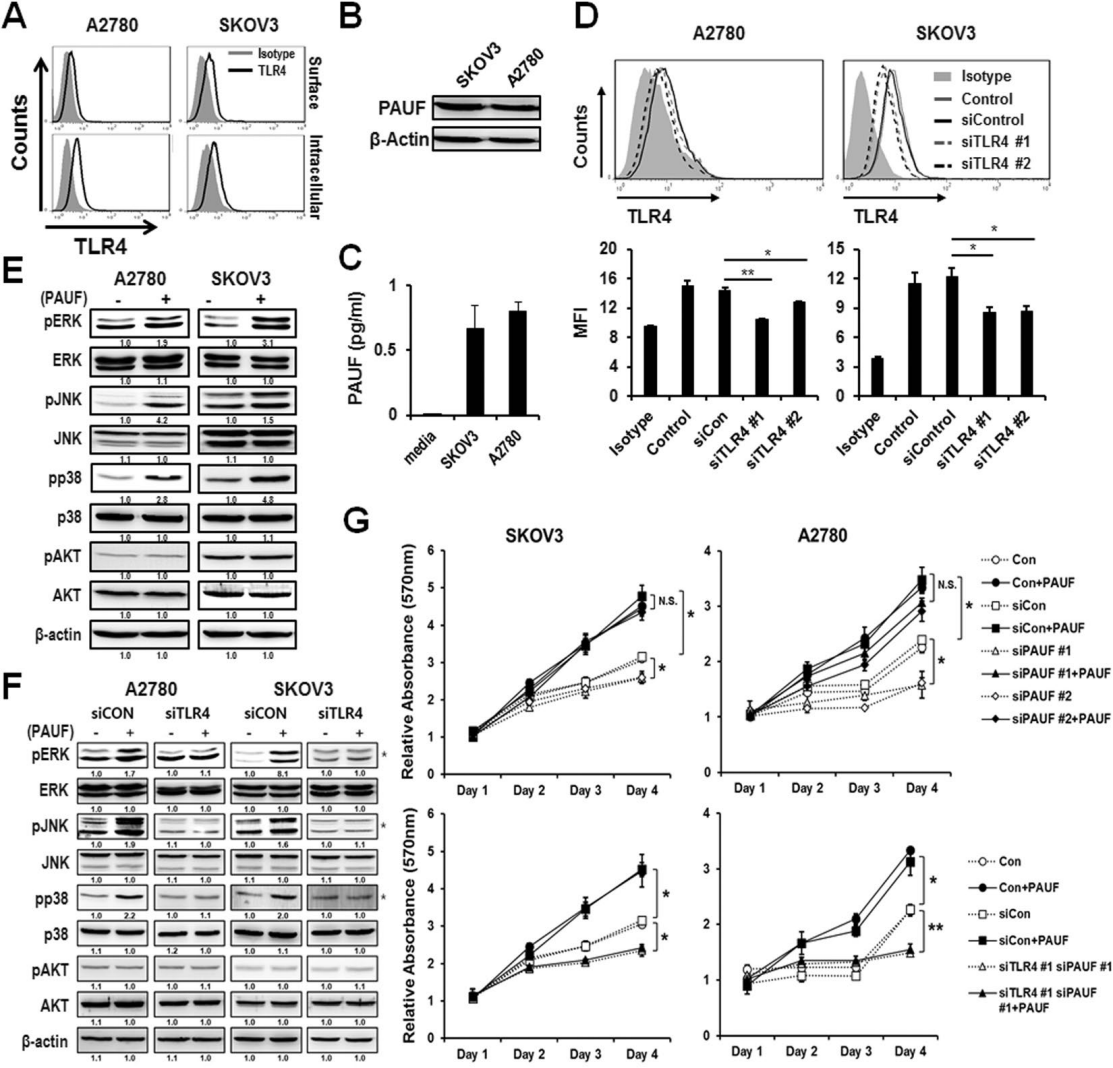
Relationship between PAUF and TLR4 expression in ovarian cancer cell lines. (**A**) To probe surface and intracellular TLR4 expression level in SKOV3 and A2780 cells, PE-conjugated TLR4 antibody was confirmed and used in flow cytometric analysis. (**B,C**) PAUF expression level and secretion in ovarian cancer cells (SKOV3, A2780) were detected using western blot analysis, and PAUF in the culture supernatant of cancer cells was detected using ELISA. (**D**) The expression level of intracellular TLR4 was assessed by flow cytometric assays. TLR4 expression was decreased significantly in both cells after transfection of two TLR4-siRNAs in comparison to that of control-siRNA or control. (MFI: Mean Fluorescence Intensity) (**E**) To determine activation of MAPKs (ERK, P38 and JNK) and AKT by PAUF, starved cancer cells were treated with or without recombinant PAUF (5 *μ*g) and analyzed by western blotting. (**F**) To confirm MAPK activation in siRNA transfected A2780 or SKOV3 cells, cells were transfected with TLR4-siRNA or control siRNA and starved for 16 hours. Cells were treated with PAUF (5 *μ*g) for 20 min and analyzed by western blotting. The number below each western blot represents the ratio of the intensity of the band over the control intensity of scramble siRNA-treated cells. (**G**) For the cell proliferation assay, control, TLR4-, or PAUF-siRNA transfected A2780 or SKOV3 cells were cultured in 96-well white plates, and cell proliferation was detected using a Cell Titer-Glo luminescence assay kit. The data shown are the means ± s.e.m. for three independent experiments. *β*-actin was used as an internal reference. *p < 0.05; **p < 0.01. The gels images were cropped and full-length gels and blots are included in the Supplementary Figure S6.

Given that PAUF activates TLR-mediated ERK signaling in pancreatic cancer, we examine its role in ovarian cancer. Since PAUF is an endogenous ligand for TLR4, we investigated whether PAUF could induce cancer cell activation and cancer proliferation via TLR4 using PAUF and TLR4 expressing ovarian cancer cell lines (A2780 and SKOV3). These two cell lines expressed TLR4 on the cell surface and intracellularly, as shown in Fig. 1A, and also expressed and secreted PAUF (Fig. 1B and C). For the knockdown of TLR4 in these cells, two kinds of TLR4 siRNAs (Sigma, MO) were transiently transfected into cells, and the TLR4 expression level was evaluated using FACS analysis and western blotting (Supplementary Fig. S1A). After 48 h post-transfection, TLR4 expression level was downregulated in all siRNA-transfected cells compared to control siRNA-transfected cells (Fig. 1D). To confirm ovarian cell activation by PAUF, starved A2780 and SKOV3 cells were treated with recombinant PAUF, and intracellular signaling cascades that are frequently important during tumor progression were detected using western blotting. Treatment of SKOV3 and A2780 cells with recombinant PAUF induced rapid activation of ERK, c-Jun N-terminal kinase (JNK), and p38 but not AKT (Fig. 1E). However, after transfection with TLR4 siRNA, activation of ERK, JNK, and p38 was reduced (Fig. 1F and Supplementary Fig. S2). The effect of silencing PAUF or TLR4 on cell proliferation was assessed in transfected A2780 and SKOV3 cells after evaluation of TLR4 and PAUF expression level by western blotting (Supplementary Fig. S1). Cell proliferation was significantly (*p* < 0.05) reduced in groups transfected with silencing siRNAs of PAUF or/and TLR4 compared to the group transfected with non-silencing control siRNA in both cell lines (Fig. 1G and Supplementary Fig. S3). The effect of recombinant PAUF treatment was investigated in transfected SKOV3 and A2780 cells with silencing siRNAs of PAUF or/and TLR4 to confirm the role of PAUF on ovarian cancer cell proliferation. Decreased proliferation in transfected SKOV3 and A2780 cells with PAUF siRNAs was completely recovered to the level of cells transfected with control siRNA by recombinant PAUF treatments. However, PAUF treatment didn’t change the decreased levels of proliferation in SKOV3 and A2780 cells transfected with a combination of both TLR4 and PAUF siRNAs. These findings demonstrate that PAUF is one of the critical factors which promote ovarian cancer cell proliferation, and TLR4 is associated with the proliferation mechanism mediated by PAUF. Collectively, our results indicate that PAUF acts on ovarian cancer cells in an autocrine and a paracrine manner to induce intracellular signaling cascades that are involved in tumor progression.

### High expression of PAUF and TLR4 is associated with advanced tumor phenotype

**Figure 2:**
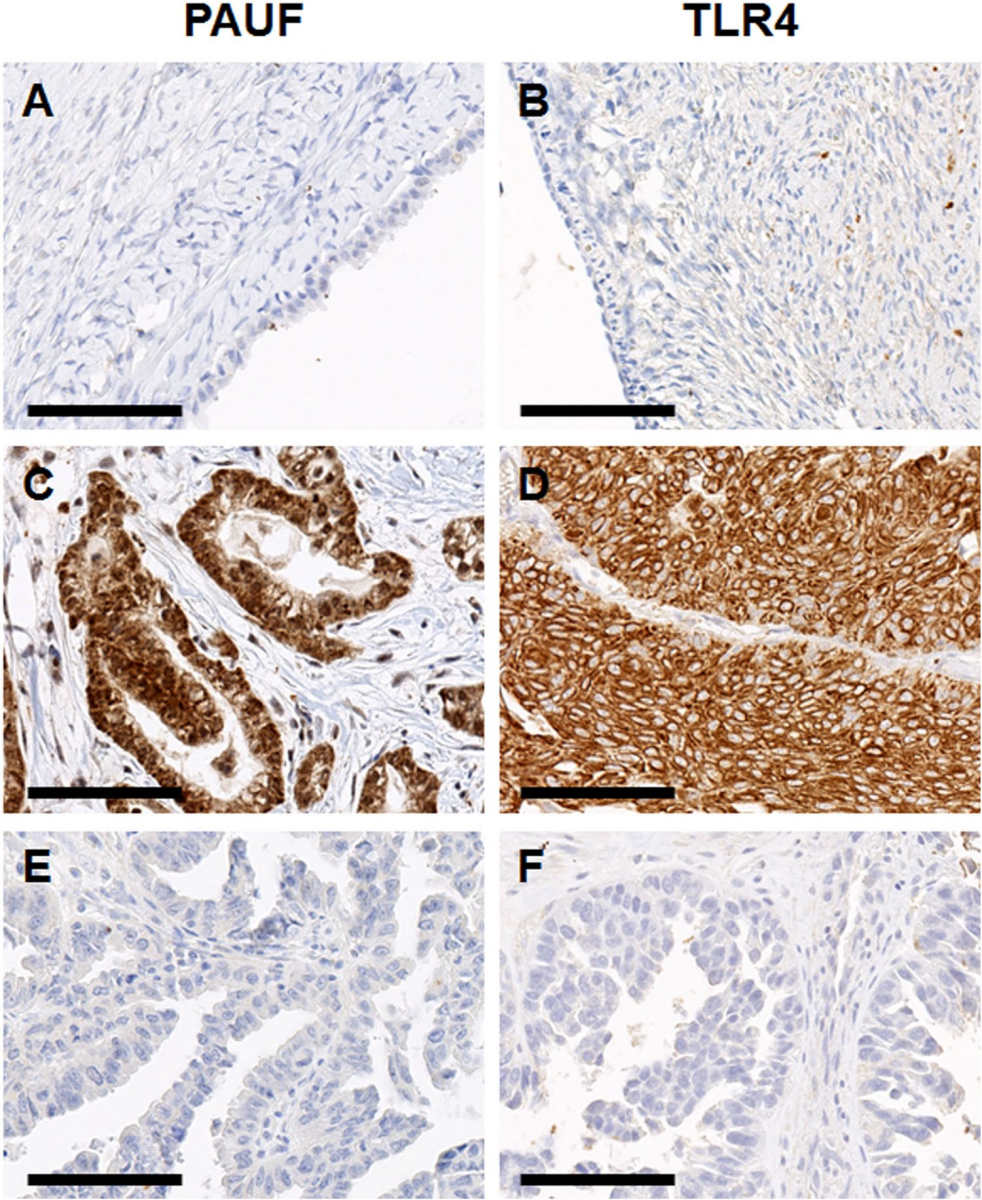
Representative images of immunohistochemical staining of pancreatic adenocarcinoma upregulated factor (PAUF) and toll-like receptor 4 (TLR4) in human ovarian cancer specimens. The top row (**A**,**B**) represents normal ovarian tissue, middle row (**C**,**D**) shows immunoreactivity of ovarian carcinomas and the bottom row (**E**,**F**) indicates immunonegativity of ovarian caricinoma. The stromal cells of normal ovarian tissue show immunonegativity for PAUF and TLR4. The scale bar represents 100 *μ*m.

**Figure 3:**
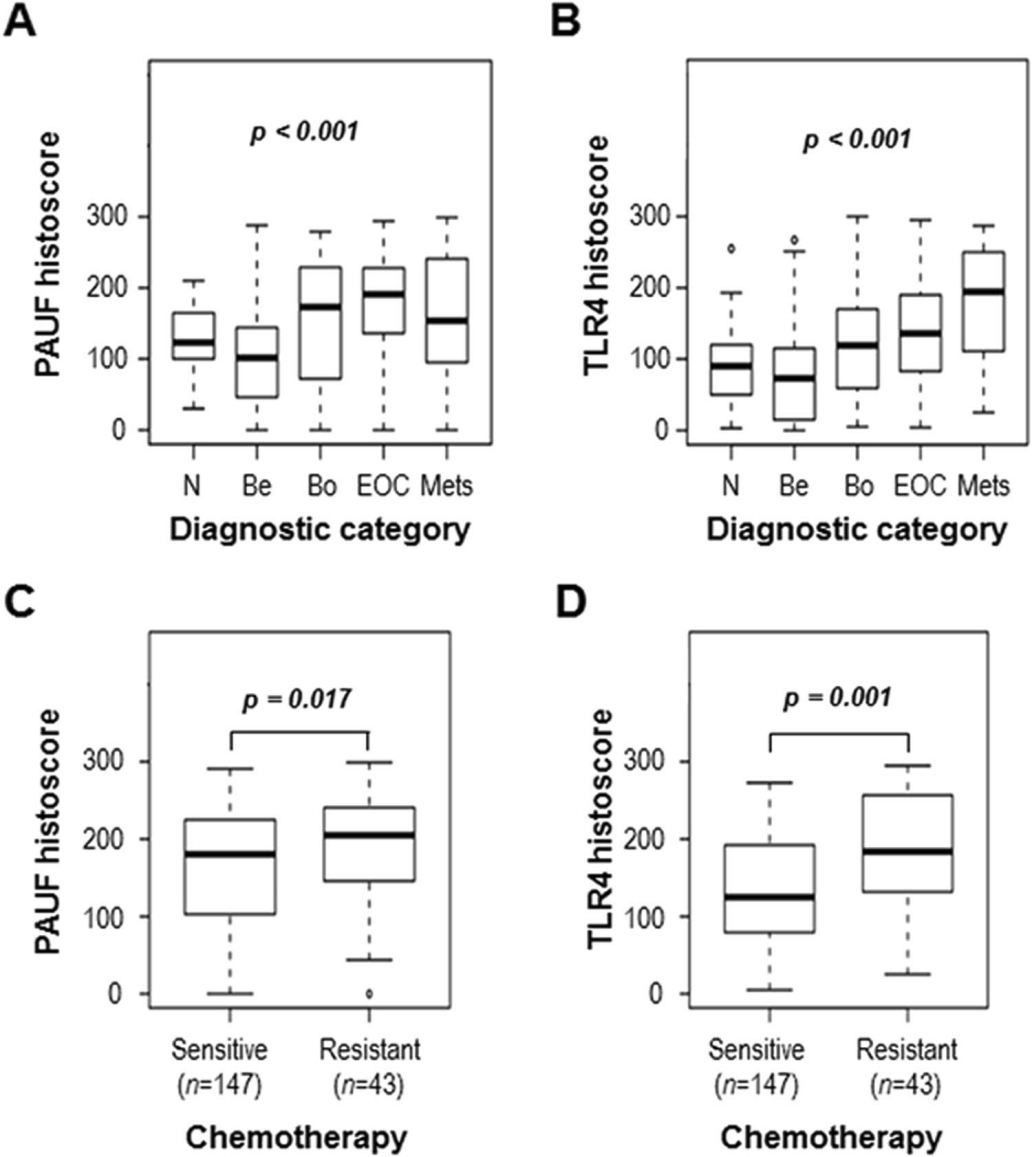
Expression of PAUF and TLR4 in patients with epithelial ovarian cancer. Expression of PAUF and TLR4 was analyzed in specimens from normal tissue (*n* = 72), benign tumor (*n* = 69), borderline tumor (*n* = 62), ovarian cancer (*n* = 205), and metastatic tissues (*n* = 52). The histoscores were computed based on intensity and tissue area of positive staining. PAUF (**A**) and TLR4 (**B**) expression increased during tumor progression. PAUF and TLR4 expression was compared between specimens from chemosensitive (*n* = 147) and chemoresistant (*n* = 43) ovarian cancer. Cancer patients who experienced recurrence within 6 months after platinum and paclitaxel chemotherapy were considered resistant. PAUF (**C**) and TLR4 (**D**) expression levels were higher in chemoresistant tumors (*p* = 0.017 and *p* = 0.001, respectively).

We examined PAUF and TLR4 expression in human epithelial ovarian tissues by immunohistochemical staining. The tumor cells were positive for PAUF as a cytoplasmic pattern, whereas TLR4 showed membranous and cytoplasmic expression pattern. Representative immunohistochemistry images of PAUF and TLR4 are presented in Fig. 2. PAUF and TLR4 were more frequently expressed in carcinoma than benign or borderline tumor. Eighty-four of 182 cancers (46.2%) had high expression (cut-off value ≥ 197) of PAUF, and 91 of 182 cancers (50.0%) had high expression (cut-off value ≥ 135) of TLR4. Clinicopathological characteristics associated with PAUF and TLR4 expression are summarized in Table 1. PAUF and TLR4 expression gradually increased from benign to cancer (Fig. 3A and B and Table 1). PAUF immunoreactivity was significantly associated with high grade (*p* = 0.014), and TLR4 immunoreactivity was associated with advanced tumor stage (*p* = 0.002). In addition, PAUF and TLR4 expression levels were higher in serous tumors than in other histology (Table 1). In terms of chemosensitivity, PAUF and TLR4 expression correlated with chemoresistant tumor (*p* = 0.017 and *p* = 0.001, respectively) (Fig. 3C and D). These results indicate that high expression levels of PAUF and TLR4 are associated with more aggressive phenotypes in epithelial ovarian cancer.

**Table 1:** Correlation between PAUF and TLR4 expression with clinicopathologic characteristics of ovarian cancer.

	No	PAUF		TLR4	
		Mean [95% CI]	*p* value	Mean [95% CI]	*p* value
Diagnosis					
Normal	72	128 [118–138]	**<0.001**	88 [76–100]	**<0.001**
Benign	69	106 [84–128]		76 [58–95]	
Borderline	62	152 [129–176]		123 [101–145]	
Cancer	205	175 [165–185]		142 [132–153]	
Age					
<50	205	156 [144–169]	0.302	129 [117–140]	0.052
>50	164	165 [154–177]		146 [133–158]	
Cell type					
Serous	164	168 [158–178]	**0.003**	151 [140–161]	**<0.001**
Others	149	141 [126–156]		101 [89–113]	
Stage					
I/II	60	173 [151–194]	0.309	116 [98–134]	**<0.001**
III/IV	140	179 [167–191]		150 [137–164]	
Metastatic	52	160 [135–185]		176 [152–201]	
Grade					
1/2	93	167 [153–180]	**0.014**	142 [127–157]	0.400
3	102	190 [177–202]		151 [137–165]	
CA125					
≤35 U/ml	80	149 [130–168]	0.126	91 [77–106]	**<0.001**
>35 U/ml	226	166 [155–176]		149 [138–159]	
Chemosensitivity					
Sensitive	147	168 [156–179]	**0.017**	138 [127–149]	**0.001**
Resistant	43	193 [175–211]		181 [158–204]	

Protein expression was determined through analysis of an immunohistochemically stained tissue array, as described in the Materials and methods section.

*p* values were measured using t-test or ANOVA.

*p* values < 0.05 are marked in bold.

**Figure 4:**
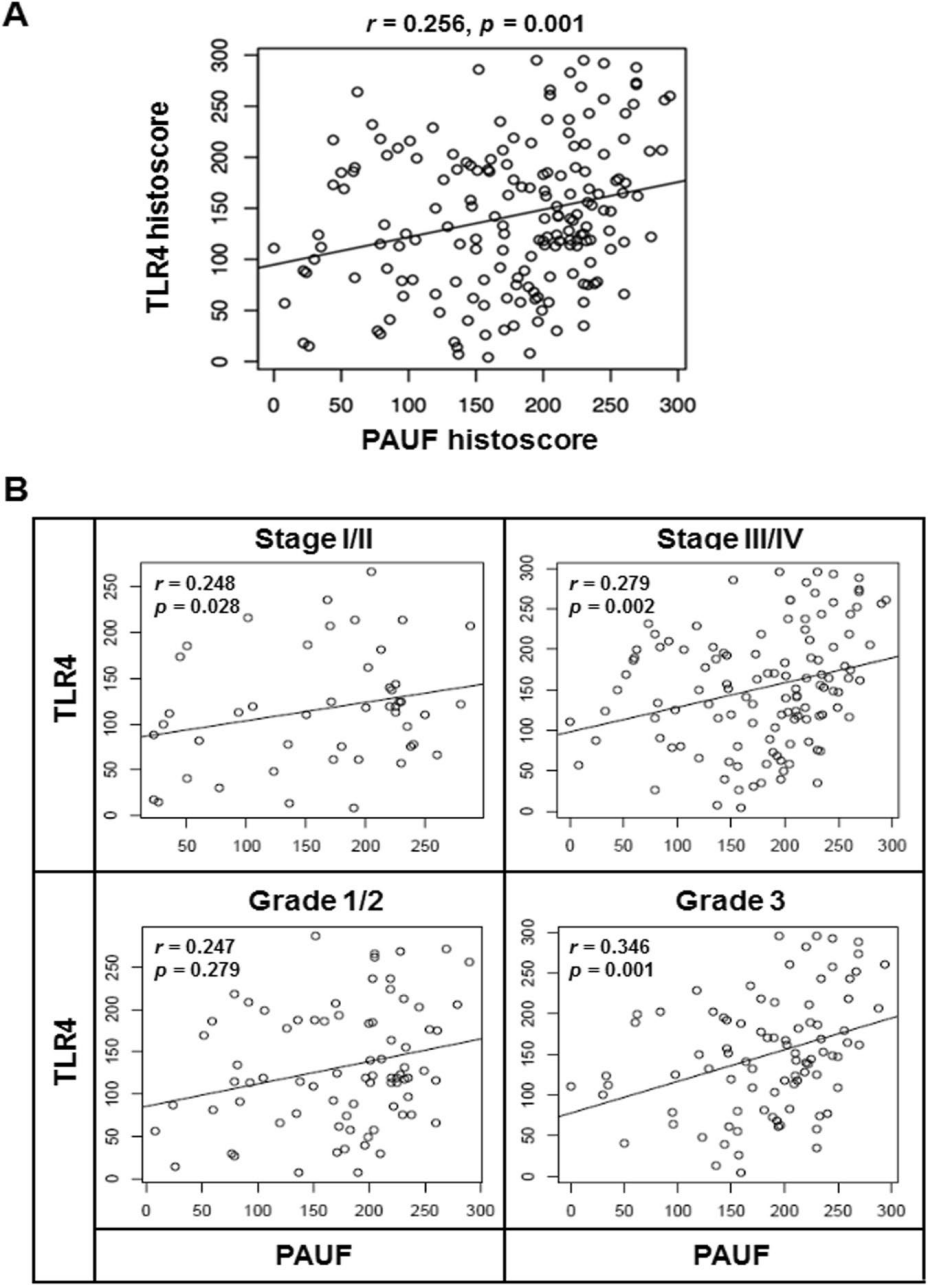
The associations between PAUF and TLR4 in patients with epithelial ovarian cancer. (**A**) TLR4 expression was positively correlated with expression of PAUF (*r* = 0.256, *p* = 0.001). (**B**) PAUF expression showed significant positive correlations with TLR4 expression in advanced stage (stage III/IV; *r* = 0.279, *p* = 0.002) and grade 3 (*r* = 0.346, *p* = 0.001).

The correlation between expression of PAUF and TLR4 was assessed in epithelial ovarian cancer and precancerous lesions. There was a weak, but statistically significant correlation between PAUF and TLR4 expression in cancer tissues (*r* = 0.256, *p* = 0.001), while there is no correlation in precancerous lesions (Fig. 4 and Supplementary Fig. S4). Furthermore, stronger correlation between PAUF and TLR4 expression was observed in advanced stages (*r* = 0.279, *p* = 0.002), grade 3 (*r* = 0.346, *p* = 0.001), chemosenstive (*r* = 0.228, *p* = 0.010) and serous (*r* = 0.271, *p* = 0.003) by subgroup analysis (Fig. 4 and Supplementary Fig. S4).

### High expression levels of PAUF and TLR4 predict shorter survival

**Figure 5:**
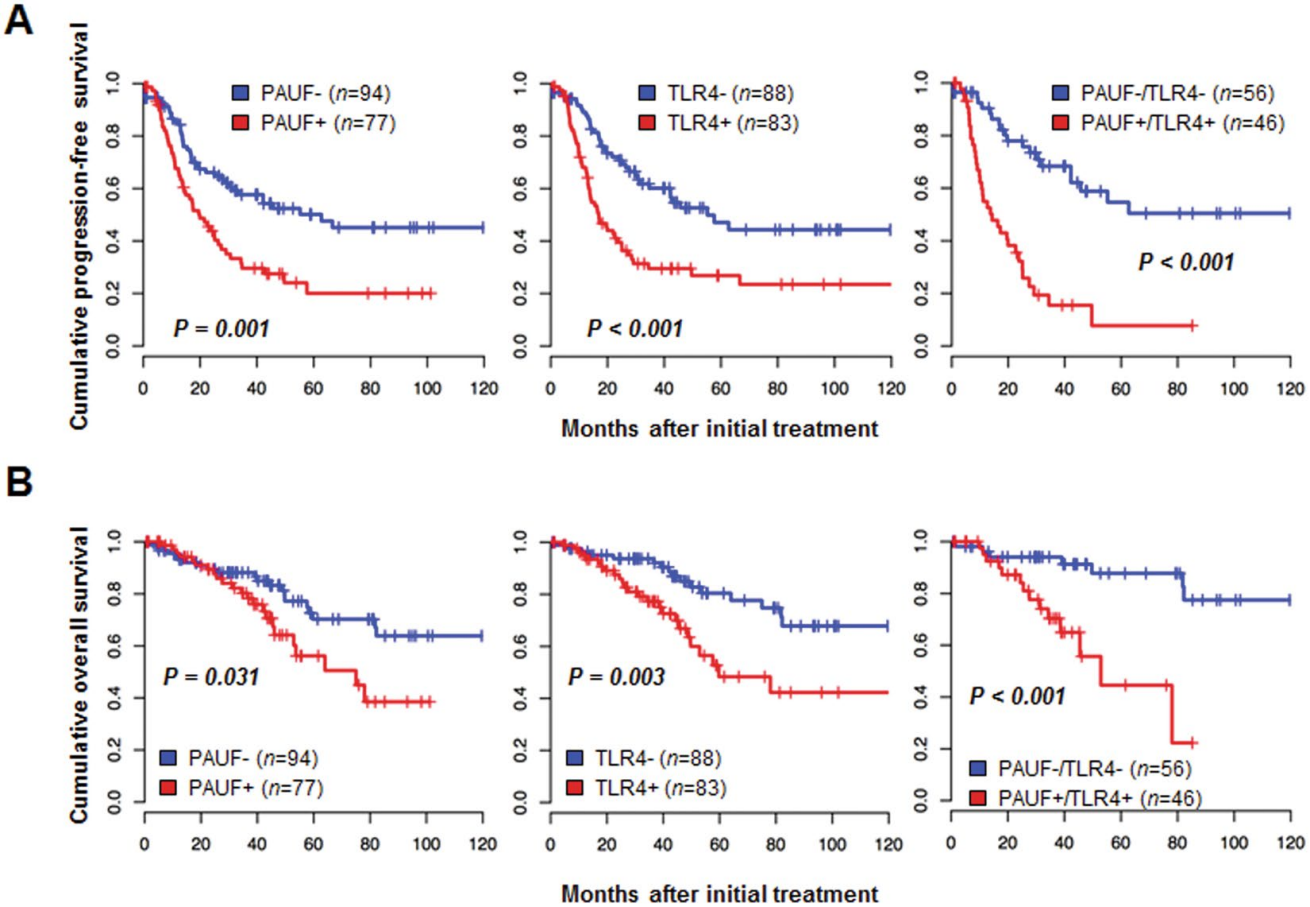
Kaplan–Meier plots of overall survival according to PAUF and TLR4 expression. (**A**) Patients with high (+) expression of PAUF or TLR4 showed worse progression-free survival than patients with low (−) PAUF or TLR4 expression (log-rank test, *p* = 0.001 and *p* < 0.001, respectively). Furthermore, patients with combined PAUF^+^/TLR4^+^ expression showed shorter progression-free survival than patients with combined PAUF-/TLR4^−^ (log-rank test, *p* < 0.001). (**B**) Patients with high (+) PAUF or TLR4 expression showed worse overall survival than patients with low (−) PAUF and TLR4 expression (log-rank test, *p* = 0.031 and *p* = 0.003, respectively). Furthermore, patients with combined PAUF^+^/TLR4^+^ expression showed shorter overall survival than patients with combined PAUF^−^/TLR4^−^ expression (log-rank test, *p* < 0.001).

We next examined the relationships between expression of each protein and patient outcomes. Kaplan–Meier plots demonstrated that patients with high expression (cut-off value ≥ 197) of PAUF displayed shorter progression-free survival (survival rate of 20.0 vs. 50.1%, *p* = 0.001) and overall survival (survival rate of 56.2 vs. 70.2%, *p* = 0.031) than patients with low PAUF expression (cut-off value < 197). The high TLR4 expression (cut-off value ≥ 135) group had also shorter survival while the low TLR4 expression (cut-off value < 135) group had longer survival in progression-free survival (survival rate of 26.8 vs. 47.1%, *p* < 0.001) and overall survival (survival rate of 48.3 vs. 80.4%, *p* = 0.003) (Fig. 5). When survival of patients with PAUF^high^/TLR4^high^ expression was compared with survival of patients with PAUF^low^/TLR4^low^ there is a significant difference in progression-free survival (survival rate of 7.7 vs. 54.7% months, *p* < 0.001) and overall survival (survival rate of 44.5 vs. 87.8%, *p* < 0.001) than patients who were PAUF^low^/TLR4^low^ expression (Fig. 5).

Multivariate analysis data using Cox proportional hazard regression were summarized in Tables 2 and 3. FIGO stage was a significant risk factor for both progression-free survival and overall survival (*p* < 0.001 and *p* = 0.045, respectively). PAUF overexpression showed independent poor progression-free survival with a hazard ratio of 2.29 (*p* < 0.001), while high TLR4 displayed poor progression-free survival and overall survival compared to low expression, as shown in Tables 2 and 3 (*p* = 0.019 and *p* = 0.038, respectively). Furthermore, the high of PAUF and TLR4 (PAUF^high^/TLR4^high^) expression was a significant risk factor for both progression-free survival [hazard ratio = 3.81 (95% CI, 1.98–7.30), *p* < 0.001] and overall survival [hazard ratio = 4.40 (95% CI, 1.48–13.06), *p* < 0.008].

**Table 2:** Univariate and multivariate analyses for progression-free survival.

Risk factor	Univariate		Multivariate	
	Hazard ratio [95% CI]	*p* value	Hazard ratio [95% CI]	*p* value
Age (>50)	1.64 [1.09–2.48]	**0.018**	1.51 [0.96–2.38]	0.076
FIGO stage (III/IV)	6.83 [3.15–14.79]	**<0.001**	5.98 [2.58–13.89]	**<0.001**
Cell type (others vs. serous)	0.38 [0.23–0.65]	**<0.001**	0.66 [0.37–1.18]	0.157
Grade (3 vs. 1/2)	1.82 [1.18–2.79]	**0.006**	1.84 [1.16–2.91]	**0.009**
PAUF ^High^	2.03 [1.34–3.08]	**<0.001**	2.29 [1.46–3.61]	**<0.001**
TLR4 ^High^	2.22 [1.46–3.38]	**<0.001**	1.74 [1.09–2.77]	**0.019**
PAUF ^High^/TLR4 ^High^	4.27 [2.39–7.63]	**<0.001**	3.81 [1.98–7.30]	**<0.001**

FIGO, International Federation of Gynecology and Obstetrics.

*p* values < 0.05 are marked in bold.

**Table 3:** Univariate and multivariate analyses for overall survival.

Risk factor	Univariate		Multivariate	
	Hazard ratio [95% CI]	*p* value	Hazard ratio [95% CI]	*p* value
Age (>50)	2.42 [1.26–4.63]	**0.008**	2.34 [1.16–4.69]	**0.017**
FIGO stage (III/IV)	4.20 [1.49–11.8]	**0.007**	2.91 [1.02–8.27]	**0.045**
Cell type (others vs. serous)	0.33 [0.14–0.79]	**0.012**	0.58 [0.24–1.40]	0.224
Grade (3 vs. 1/2)	1.88 [1.01–3.52]	0.048	2.05 [1.06–3.96]	**0.033**
PAUF ^High^	1.92 [1.05–3.51]	**0.034**	1.83 [0.97–3.44]	0.062
TLR4 ^High^	2.53 [1.34–4.8]	**0.004**	2.08 [1.04–4.15]	**0.038**
PAUF ^High^/TLR4 ^High^	5.29 [1.97–14.19]	**0.001**	4.40 [1.48–13.06]	**0.008**

FIGO, International Federation of Gynecology and Obstetrics.

*p* values < 0.05 are marked in bold.

## Discussion

In this study, we demonstrated that PAUF enhanced cancer cell growth through ERK, JNK, and p38 activation using two ovarian cancer cell lines (A2780 and SKOV3). Selective knockdown of TLR4 using siRNA significantly reduced the activation levels of ERK, JNK, and p38 and the growth rates of A2780 and SKOV3 cells. Subsequently, we investigated the prognostic significance of PAUF and TLR4 expression in epithelial ovarian tumors. PAUF and TLR4 protein expression was increased during carcinogenesis. Notably, overexpression of PAUF and TLR4 correlated with aggressive tumor phenotypes, including chemoresistance. Patients with PAUF and TLR4 overexpression had shorter median progression-free survival and overall survival. These findings suggest that PAUF participates in the progression of ovarian cancer via TLR4 signaling that activates ERK, JNK, and p38. Therefore, the assessment of PAUF and TLR4 expression can potentially serve as a new prognostic indicator predicting survival time, and can be helpful in management of patients with ovarian cancer.

The engagement of TLR4 signaling in cancer was revealed by previous studies. TLR4 signaling is upregulated in numerous ovarian epithelial cancers, and the level of expression correlates with increased cancer progression and chemoresistance to paclitaxel^26–28^. Recently, Luo *et al*. have suggested that TLR4 might stimulate serous ovarian carcinoma initiation, progression, and chemoresistance^29^. The poor outcome and chemoresistance of ovarian cancer patients with TLR4 overexpression in our study are consistent with those previous studies. However, previous data on the clinical significance of PAUF expression in ovarian cancer were limited. Kim *et al*. found positive staining for PAUF more frequently in mucinous adenocarcinoma than in mucinous cystadenoma and mucinous borderline tumor and showed that patients with PAUF-positive cancer tended to have shorter survival. They suggested that PAUF might be a prognostic marker for patients with an ovarian mucinous tumor^30^. Our data demonstrated that PAUF and TLR4 expression were increased from benign to advanced tumor (Fig. 3), and their correlation coefficient was increased in advanced stages (stage III/IV) and grade 3 compared to early stages (stage I/II) and grade 1/2 cancer specimens (Fig. 4). These data suggest that the PAUF and TLR4 is closely linked in the process of epithelial ovary cancer. The potential of PAUF/TLR4 as a prognostic marker was first investigated in the present study.

Ovarian cancer remains a very difficult disease to treat as most patients present at an advanced stage. Most patients respond to initial anticancer therapy but experience tumor recurrence within 3 years^31^. Chemoresistance is one of the major clinical problems compromising the successful treatment of ovarian cancer. Several mechanisms have been proposed to be involved in drug resistance, including decreased drug accumulation, alteration of drug transport, increased drug tolerance, and increased DNA repair activity^32–34^. Here, we propose that the PAUF/TLR4 signaling pathway is one of the mechanisms involved in drug resistance and is associated with poor prognosis of patients.

PAUF was previously reported as an endogenous ligand for TLR4, and activates the mitogen-activated protein kinase pathway in THP-1 cells. PAUF also activates the TPL2/MEK/ERK signaling pathway, resulting in an increase of AP-1 regulated gene expression and promoting escape from innate immune surveillance and tumor growth^25^. In addition, Kang *et al*. showed that PAUF induces activation and maturation of dendritic cells by stimulating the TLR signaling pathway^35^. They showed that treatment with PAUF or lipopolysaccharide (LPS) induces production of IL-23 in maturing dendritic cells in a TLR4-dependent manner. Recently the change of tumor microenvironment by activation of TLR4 was reported in different types of cancer including diffuse large B-cell lymphoma^36^, breast^37^ and prostate^38^ cancers. It postulates that the delicate balance of PAUF/TLR activation in the tumor microenvironment in different cell types helps to shape the inflammatory profile and outcome of tumor growth or regression. Song *et al*. reported that PAUF enhances accumulation of tumor-infiltrating Myeloid-derived suppressor cells and its immune suppressive function via TLR4-mediated signaling pathway in PAUF-overexpressing tumor cell-injected mice^39^. With further studies, specific activation or repression of PAUF/TLRs can be harnessed to offer novel immunotherapies or adjuvants to traditional chemotherapy for some ovarian cancer patients.

In summary, we showed that PAUF^high^ and TLR4^high^ expression is associated with aggressive tumor phenotypes and is an independent prognostic factor for progression-free survival, while TLR4^high^ and PAUF^high^/TLR4^high^ is poor prognostic factor for overall survival in epithelial ovarian cancer. Furthermore, we demonstrated that PAUF could induce cancer cell activation and cancer proliferation via ERK, JNK, and p38 activation. Further characterization of the mechanism of PAUF in chemoresistance will aid the development of novel treatments for epithelial ovarian cancer.

## Methods

### Patients and tumor samples

A total of 72 normal epithelial tissues, 69 benign tumor tissues, 62 borderline tumors, 205 epithelial ovarian cancers and 52 metastatic tumors were obtained from patients who underwent surgical treatment at Gangnam Severance Hospital between 1996 and 2010 and the Korea Gynecologic Cancer Bank (NRF-2012M3A9B8021800). All procedures were conducted in accordance with the Declaration of Helsinki. All study participants provided written informed consent tissue samples with the Institutional Review Board of Samsung Medical Center (approval no. 2015-07-122; Seoul, South Korea).

Ovarian cancers were classified based on the International Federation of Gynecology and Obstetrics (FIGO) staging system and the WHO grading system. All patients were treated with maximal debulking surgery, followed by combination treatment with paclitaxel/carboplatin. The clinicopathological features are summarized in Table 1. After platinum based chemotherapy, follow-up examinations were done every 3 months for the first 2 years, 6 months for the next 3 years and subsequent annual checkups. Progression-free survival was evaluated from the date of surgery to the period of recurrence/progression or the time of the last follow-up visit. Overall survival was assessed from the date of surgery to patient death, or the date of last contact, for living patients.

### Immunohistochemistry

Tissue cylinders of 1.0 mm diameter were extracted from the most representative areas of donor blocks and transplanted into recipient blocks using a tissue arrayer (Beecher Instruments, Inc., Silver Spring, MD). According to the block, 2–3 punches from each patient were included in the TMA, and the final expression values were averaged. For the assessment of PAUF and TLR4 expression, 5-*μ*m TMA sections were used for immunohistochemical staining as described previously^23^. Antigen recovery was performed in heat-activated antigen retrieval buffer of pH 9.0 (for PAUF) or pH 6.0 (for TLR4) (Dako, Carpinteria, CA). For TLR4, additional protein blocking (Dako) was applied for 15 min. The TMA slides were incubated at room temperature with anti-PAUF mouse monoclonal antibody (clone no. 817310; R&D Systems, Minneapolis, MN) at 1:100 dilution for 2 h, mouse monoclonal anti-TLR4 antibody (NB100–56566: Novus Biologicals, Littleton, CO) at 1:250 dilution for 1 h. The sections were incubated with with EnVision^+^ Dual Link System-HRP (Dako) for 30 min, visualized with DAB^+^ (3, 3′-Diaminobenzidine; Dako) for 10 min, and counterstained with hematoxylin. Negative controls including mouse immunoglobulin G (IgG) isotype control and omission of the primary antibody were performed (Supplementary Fig. S5). Pancreatic adenocarcinoma and lymph nodes were used as positive controls.

Quantitative digital image analysis of immunohistochemical stains was performed using Visiopharm integrator system (VIS) for Windows 7, version 4.5.1.324 (Visiopharm, Hoersholm, Denmark) as described previously^40^. In brief, slides were scanned using bright field imaging at × 20 magnification (NanoZoomer 2.0 HT: Hamamatsu Photonics, Hamamatsu City, Japan). The brown-colored (3, 3′-Diaminobenzidine) staining intensity was calculated using a predefined algorithm setting. The overall protein expression was expressed as mean value of histoscore, which is the multiplication of the intensity score (0–3) and percentage of stained cells, with a maximum of 300^40^.

### ELISA

SKOV3 and A2780 cells were purchased from ATCC (Manassas, VA). A total of 3 × 10^5^ SKOV3 and A2780 cells were cultured in opti-MEM media (Gibco, Waltham, MA) for 48 h. Culture supernatant of cancer cells was used for the detection of PAUF by ELISA^27^. Plates were coated with 4E6 capture antibodies (5 μg/ml) for 16 h at room temperature and then incubated with the supernatant of cultured cancer cells for 2 h. 11G6 detection antibodies (250 ng/ml) were added for 90 min at 37 °C, and then streptavidin-HRP (1:5000) was added for a further 30 min at 37 °C. PAUF expression level was detected at 450 nm.

### Flow cytometry and intracellular staining

Cultured SKOV3 and A2780 cells were washed and stained with PE-conjugated anti-TLR4 antibodies (eBioscience, San Diego, CA) for confirmation of surface staining or washed, fixed, permeabilized using a BD cytofix/cytoperm Plus kit (BD Bioscience, San Jose, CA), and stained with PE-conjugated TLR4 antibodies for confirmation of intracellular TLR4 level. Cells were analyzed on a FACSCalibur using CELL Quest software (BD Bioscience).

### Transfection

A2780 (4 × 10^5^) and SKOV3 (2 × 10^5^) cells were cultured in 6-well plates. Cells were cultured in opti-MEM for 1 h and then incubated with 400 pmol of control siRNA (Cosmo Genetech, Seoul, Korea) (Sense: 5′-UCCCUGGGUAUUCCCACCUAAGGCU-3′, Antisense: 5′-AGCCUUAGGUG GGAAUACCCAGGAA-3′)^21^, TLR4 #1 siRNA (Sense: 5′-GGGCUUAGAACAACUAGAATT-3′, Antisense: 5′-UUCUAGUUGUUCUAAGCCCTT) (Sigma, NM_138554, Missouri 63103, USA), TLR4 #2 siRNA (Sense: 5′-CGAUGAUAUUAUUGACUUA-3′, Antisense: 5′-UAAGUCAAUAA UAUCAUCG-3′), PAUF #1 siRNA (Sense: 5′-UCCCUGGGUAUUCCCACCUAAGGCU-3′, Antisense: 5′-AGCCUUAGGUGGGAAUACCCAGGAA-3′) or PAUF #2 siRNA (Sense: 5′-AAAUAGAAAUAGCGGUCCUUGCUGG-3′, Antisense: 5′-CCAGCAAGGACCGCUAUUUCUA UUU-3′, Cosmo Genetech) for 8 h with lipofectamine RNAi max (Invitrogen, Waltham, MA). Cells were washed and cultured for 48 h. One more transfection was conducted, and cells were incubated for 18 h.

### Western blot analysis

To confirm intracellular PAUF expression level or cell signaling activation after siRNA transfection and treatment with recombinant PAUF, A2780 and SKOV3 cells were cultured in 100-mm culture plates. After transfection with control, PAUF #1, PAUF #2, TLR4 #1 or TLR4#2 siRNA, cells were starved in 6-well plates for 16 h and incubated with or without recombinant PAUF protein (5 µg/ml) for 20 min. Cells were washed, collected, centrifuged, and lysed with protein extraction RIPA solution (50 mM Tris-Cl [pH 8.0], 150 mM NaCl, 1 mM phenylmethylsulphonyl fluoride [PMSF], 0.1% sodium dodecyl sulfate [SDS], 1% Nonidet P-40 [NP-40], and 0.5 mM EDTA; Elpis Biotech, Daejeon, Korea) for 1 h on ice. Protein concentrations were determined using a Bradford protein assay kit (Pierce, Waltham, MA). Equal amounts of protein were solubilized in SDS-PAGE loading buffer (250 mM Tris-HCl, pH 6.8, 0.5 M DTT, 10% SDS, 0.5% bromophenol blue, 50% glycerol), subjected to electrophoresis, and transferred to PVDF membranes (Traub & Co., Basel, Switzerland). Membranes were probed with anti-rabbit phosphor-ERK (Cell Signaling Technology, #9102, USA), total ERK (Cell Signaling Technology, #9101), phosphor-JNK (Cell Signaling Technology, #9251), total JNK (Cell Signaling Technology, #9251), phosphor-p38 (Cell Signaling Technology, #4511), total p38 (Cell Signaling Technology, #9212), phosphor-AKT (Cell Signaling Technology, #9271), total AKT (Cell Signaling Technology, #9272), PAUF (R&D Systems, MAB7777, USA), or anti-mouse β-actin (Santa Cruz Biotechnology, sc-47778, USA) antibodies 1000 diluted in 5% BSA and then incubated with horseradish peroxidase (HRP)-conjugated goat anti-rabbit (Enzo Life Sciences, New York, USA) or mouse IgG (Cell Signaling Technology) secondary antibodies. After addition of chemiluminescent HRP Substrate (Millipore, Billerica, USA), immunoreactive bands were visualized by an enhanced chemiluminescence reaction. The intensity of bands was determined using ImageQuant software (Ver. 5.2, GE Healthcare, Piscataway, USA). The ratio is the intensity ratio compared to the western blot intensity of scramble siRNA-treated cells.

### Cell proliferation

SKOV3 (2 × 10^3^) and A2780 (4.5 × 10^3^) cells untransfected or transfected with control, TLR4#1, TLR4 #2, PAUF #1 or PAUF #2 siRNA were cultured with PAUF protein (1 μg/ml) day after day in 96-well white plates (SPL Life Sciences, Pocheon, Korea). Cell proliferation was measured from one to four days using a Cell Titer-Glo luminescence assay kit (Promega, Madison, WI).

### Statistical analysis

Statistical analysis was performed with R statistical software version 3.4.1. Student’s t test or ANOVA test was used to test possible associations between immunohistochemical expression of the proteins and clinicopathological factors. Spearman’s rank correlation coefficient was used to assess the correlations between expressions of each protein. For survival analysis, samples were categorized as positive or negative using the cutoff values showing the greatest discriminative power in a Cox model of progression-free survival (R package: survMisc). Kaplan-Meier survival curves were plotted and the log rank test was used to compare survival between groups. Survival distributions were plotted using the Kaplan–Meier method, and the relationship between survival and each parameter was analyzed with the log-rank test. A multivariate Cox proportional hazards model was used to identify independent prognostic factors of survival. *P*-values of 0.05 or less were considered statistically significant.

## Electronic supplementary material

Supplementary figures S1-S8.
