# Supplementary material for: Elevated expression of pancreatic adenocarcinoma upregulated factor (PAUF) is associated with poor prognosis and chemoresistance in epithelial ovarian cancer
Source: Sci Rep. 2018 Aug 15;8:12161. doi: 10.1038/s41598-018-30582-8 (PMC6093878; doi:10.1038/s41598-018-30582-8)

**Elevated expression of pancreatic adenocarcinoma upregulated factor (PAUF) is associated with poor prognosis and chemoresistance in epithelial ovarian cancer**

Chel Hun Choi^1,5,†^, Tae Heung Kang^2, †^, Joon Seon Song^1,6^, Young Seob Kim^2^, Eun Joo Chung^3^,

Kris Ylaya^1^, Seokho Kim^7^, Sang Seok Koh^8^, Joon-Yong Chung^1^, Jae-Hoon Kim^4,*^,

Stephen M. Hewitt^1,**^

*^1^Experimental Pathology Laboratory, Laboratory of Pathology, Center for Cancer Research, National Cancer Institute, National Institutes of Health, Bethesda, MD 20892 USA*

^2^*Department of Immunology, College of Medicine, Konkuk University, Chungju 27478, Republic of Korea*

*^3^Radiation Oncology Branch, Center for Cancer Research, National Cancer Institute, National Institute of Health, Bethesda, MD 20892, USA*

*^4^Department of Obstetrics and Gynecology, Gangnam Severance Hospital, Yonsei University College of Medicine, Seoul 06273, Republic of Korea*

*^5^Departments of Obstetrics and Gynecology, Samsung Medical Center, Sungkyunkwan University School of Medicine, Seoul 06351, Republic of Korea*

*^6^Department of Pathology, Asan Medical Center, University of Ulsan College of Medicine, Seoul 05505, Republic of Korea*

*^7^Aging Research Institute, Korea Research Institute of Bioscience and Biotechnology, Daejeon 34141, Republic of Korea*

*^8^Department of Biological Sciences, Dong-A University, Busan 49315, Republic of Korea*


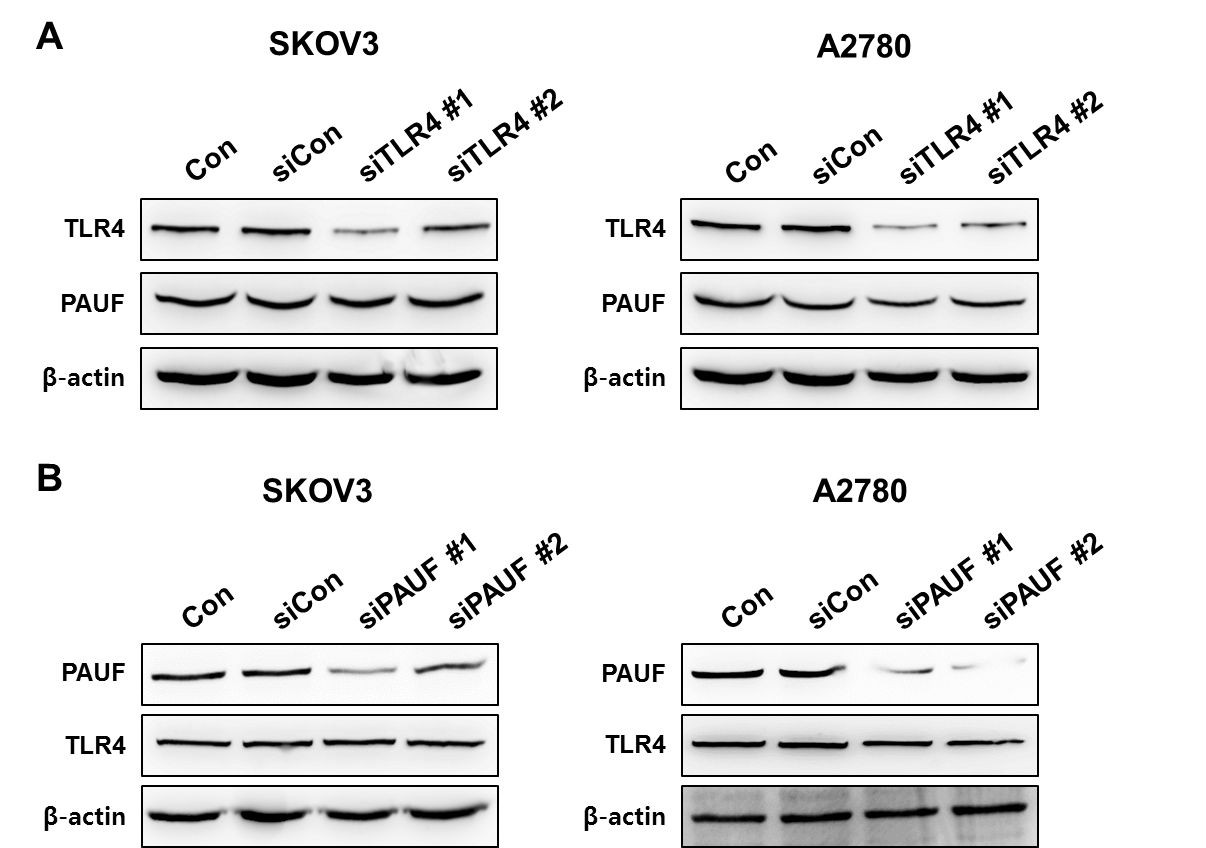
**Supplementary Figure S1. The demonstration of successful knockdown of TLR4 (A) and PAUF (B) in SKOV3 and A2780 cells. The gels images were cropped and full-length gels and blots are included in the Supplementary Figure S6.**

**Supplementary Figure S2. The confirmation of reduced MAPK activation by transfection of siTLR4 #2 in SKOV3 (A) and A2780 (B) cells. The gels images were cropped and full-length gels and blots are included in the Supplementary Figure S7.**

**
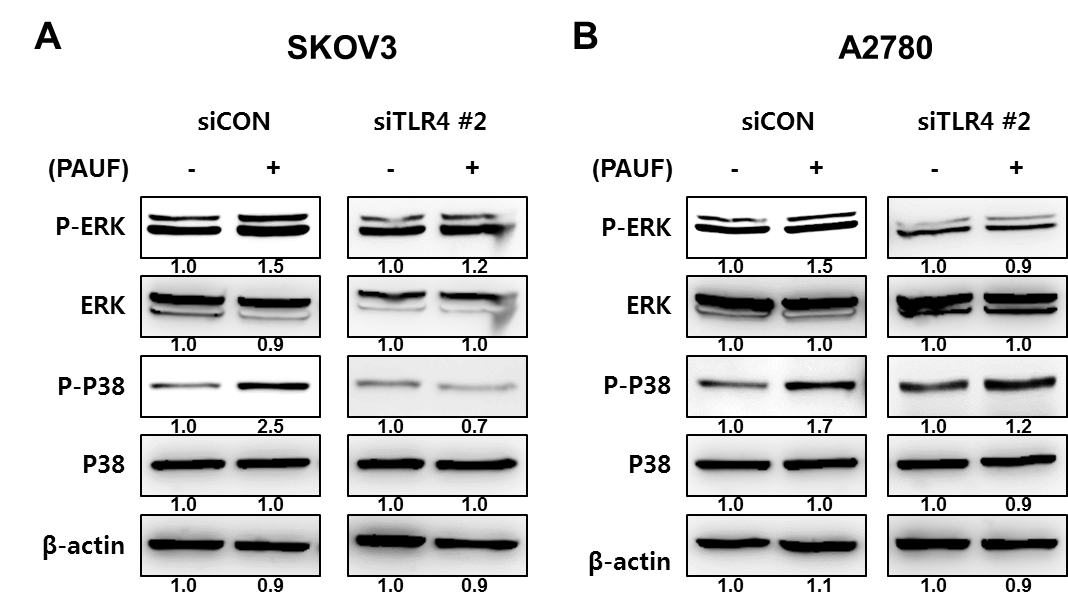
**

**Supplementary Figure S3. Proliferation assay of siCon, TLR4-, or PAUF-siRNA transfected A2780 and SKOV3 cells. *, *p* < 0.01; **, *p* < 0.001.**

**
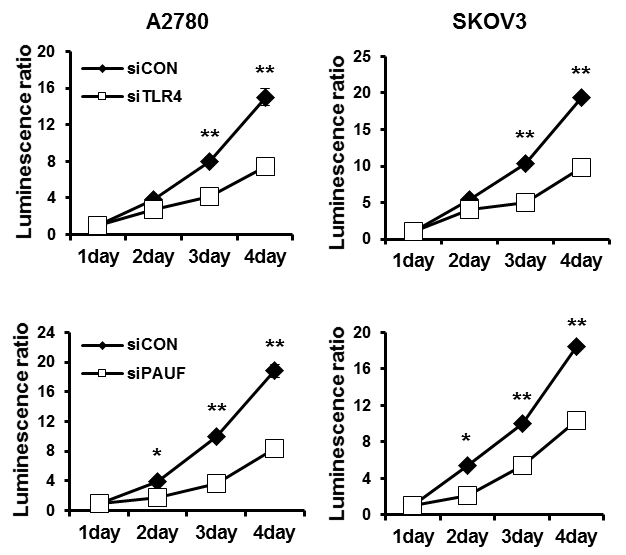
**

**Supplementary Figure S4. PAUF and TLR4 expression in human precancerous and ovarian cancer. (A) PAUF expression was not correlated with TLR 4 expression in precancerous lesions. (B) PAUF expression was positively corelated with TLR4 expression in chemosensitive and serous groups (*p*=0.010 and *p*=0.003, respectively).**

**
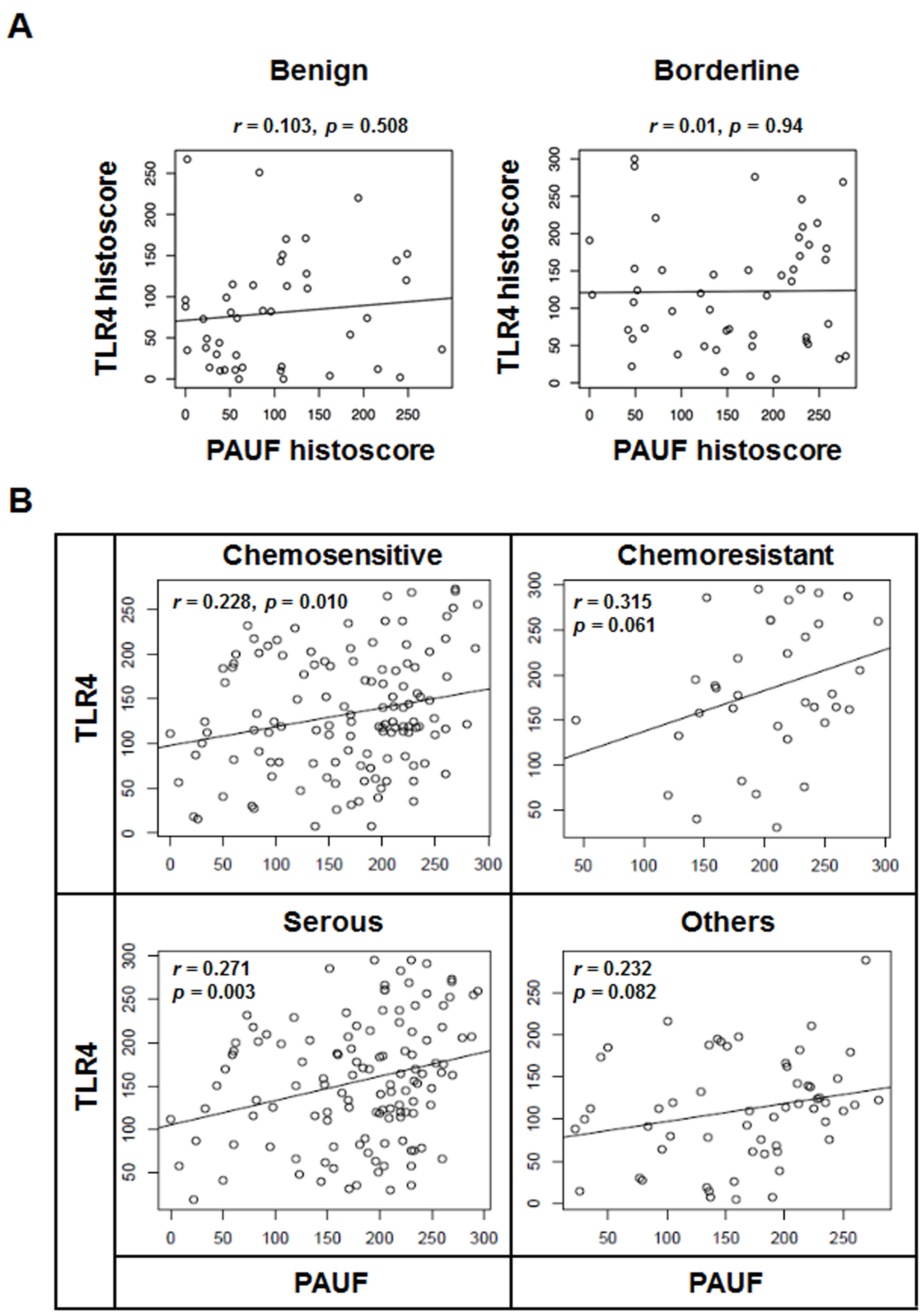
**

**Supplementary Figure S5. Immunohistochemical staining of TLR 4. Immunohistochemical staining with TLR4 in neutrophilic granulocyte (marked with arrow) of normal ovarian epithelia was used as a positive control (A). Mouse immunoglobulin G (IgG) isotype (B) and no primary antibody (C) were used as negative controls. Scale bar, 100 *µ*m.**

**
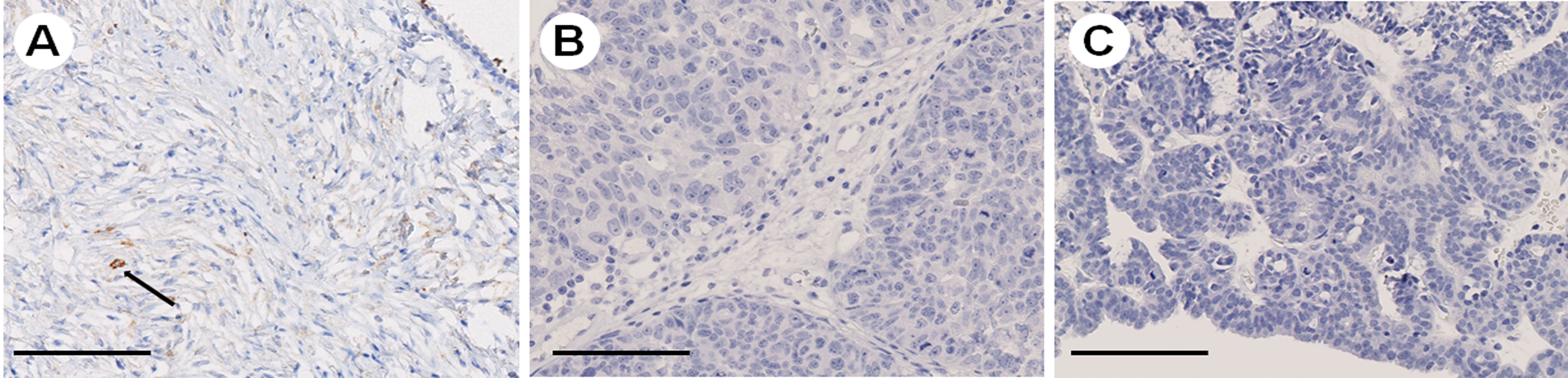
**

**Supplementary Figure S6. Full-length blots of Fig. 1**

**
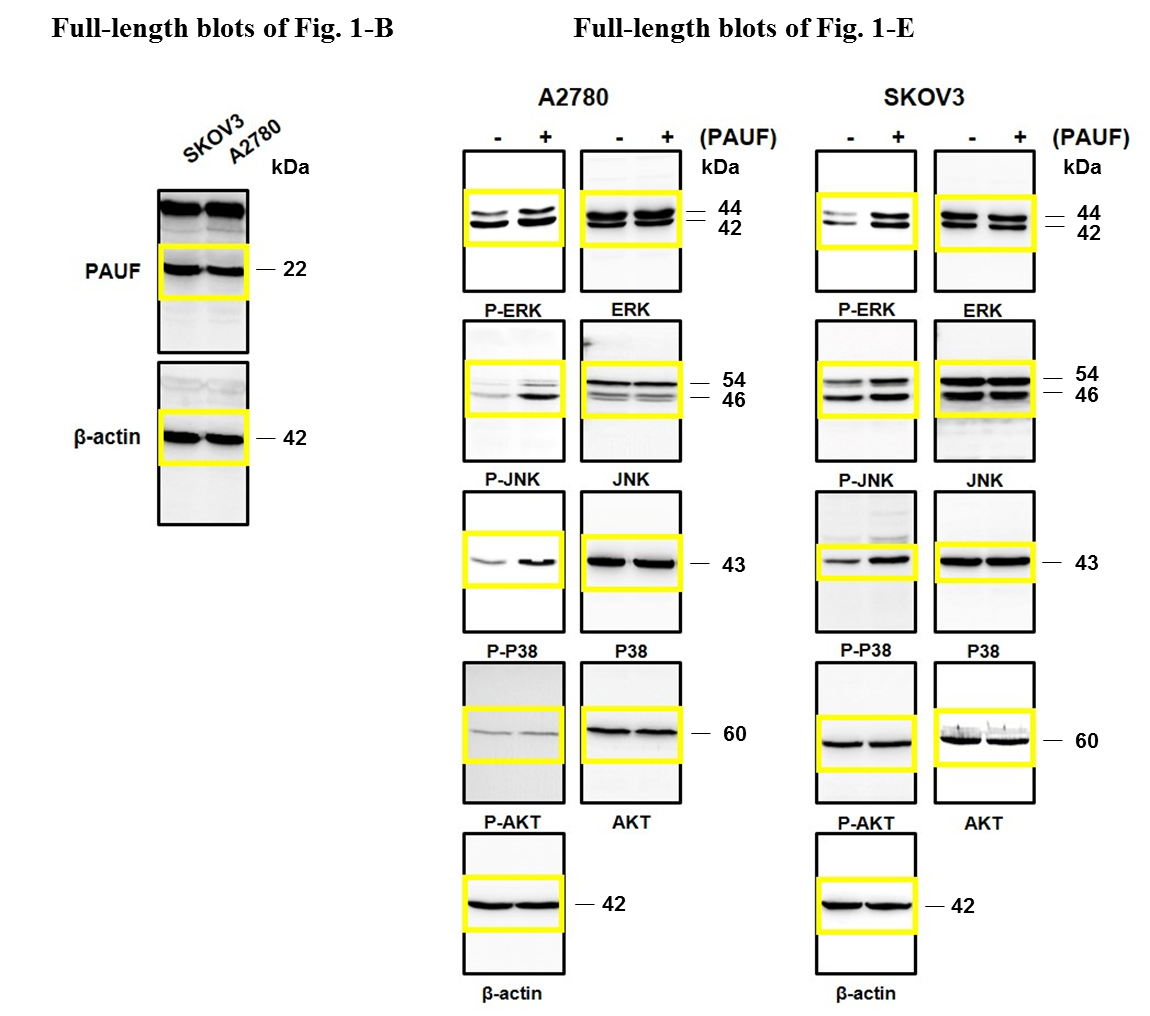
**


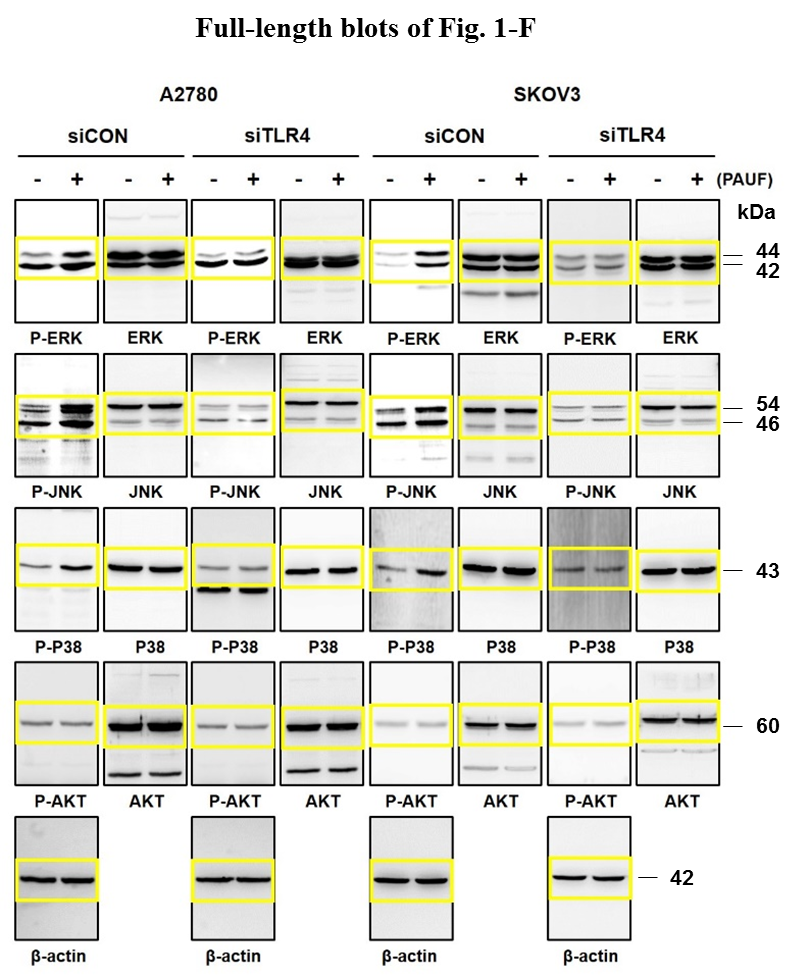


**Supplementary Figure S7. Full-length blots of Supplementary Fig. 1.**

**Full-length blots of Supplementary Fig. 1A**


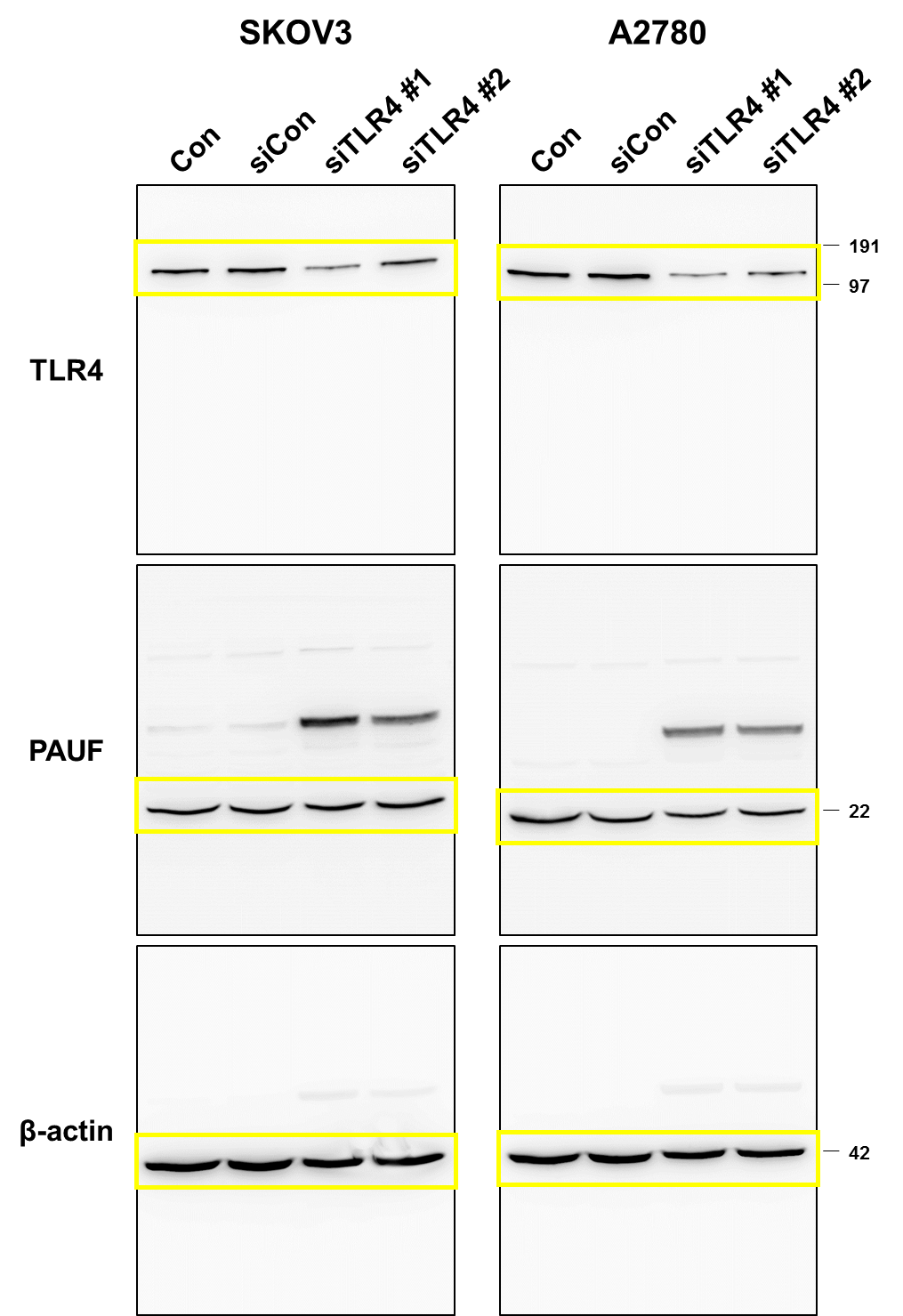


**Full-length blots of Supplementary Fig. 1B**


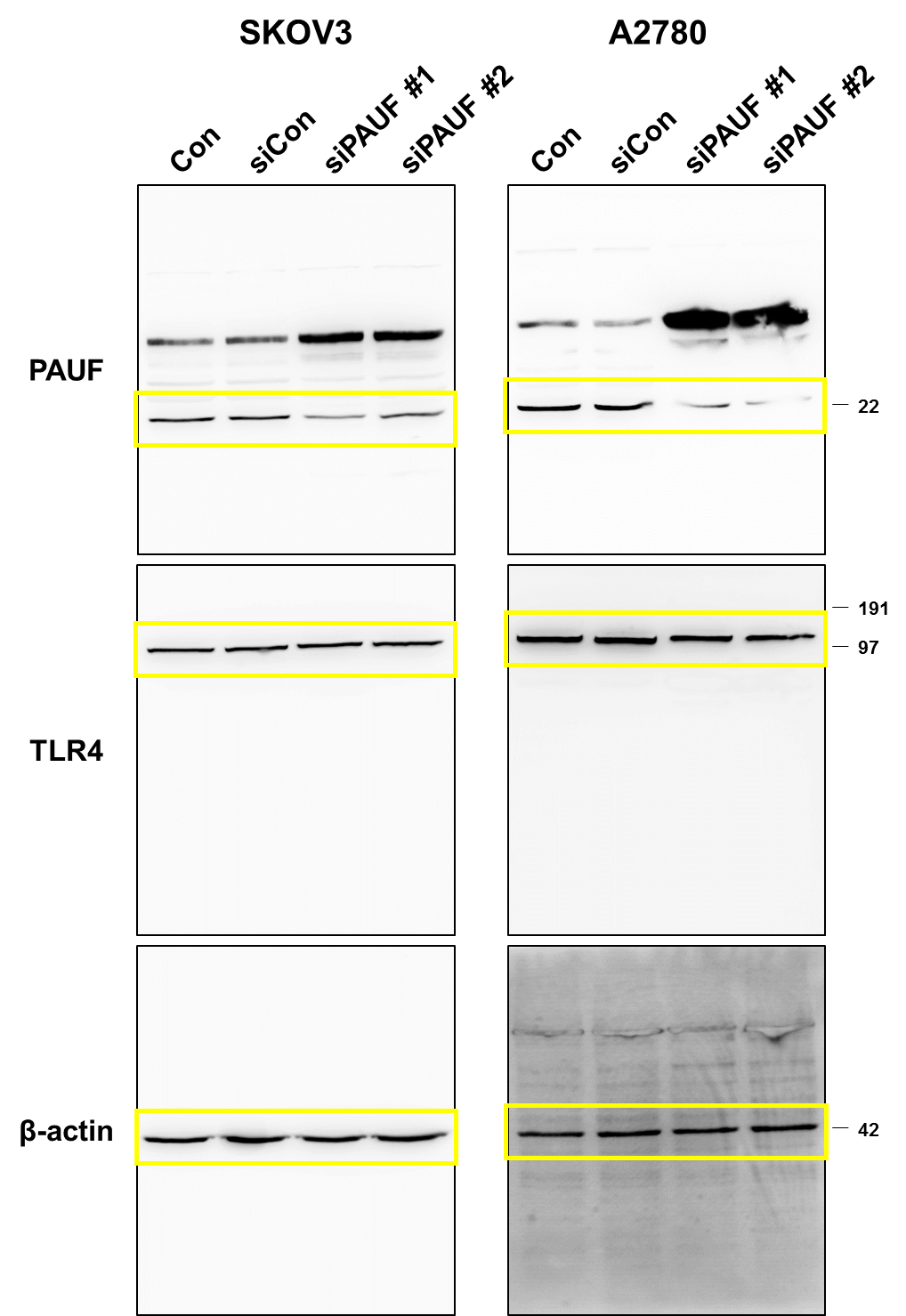


**Supplementary Figure S8. Full-length blots of Supplementary Fig. 2**


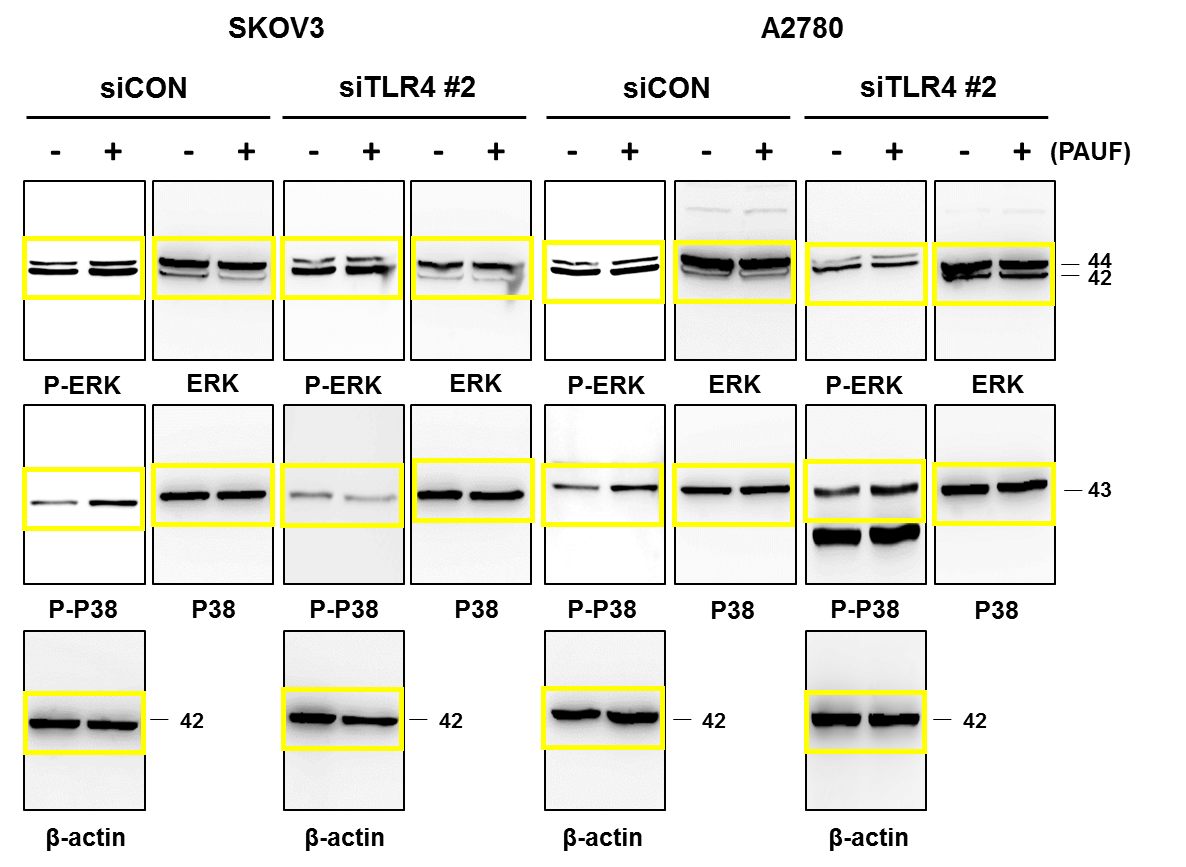

Supplement: Supplementary file 1 — Supplementary figures S1-S8. [file 41598_2018_30582_MOESM1_ESM.doc]
